# Emergency cesarean section in pregnant trauma patients presenting after motor vehicle collision

**DOI:** 10.1016/j.heliyon.2024.e38707

**Published:** 2024-09-28

**Authors:** Michelle Hough, Jeffry Nahmias, Jeffrey Santos, Lourdes Swentek, Robert Bristow, Jennifer Butler, Areg Grigorian

**Affiliations:** University of California, Irvine, Department of Surgery, Division of Trauma, Burns and Surgical Critical Care, Orange, CA, USA

## Abstract

**Background:**

Most pregnant trauma patients (PTPs) present after motor vehicle collision (MVC). The national rate and risk factors for emergency cesarean section (ECS) during the index hospitalization for pregnant trauma patients (PTPs) are unknown. We sought to investigate the national rate of ECS in PTPs presenting after MVC, hypothesizing a higher risk of ECS among those with severe injuries or elevated shock index (SI).

**Methods:**

The 2020–2021 TQIP was queried for PTPs presenting after MVC. PTPs that underwent ECS were compared to patients that did not undergo ECS. Elevated SI was defined as ≥1. Severe injury was defined by abbreviated injury scale grade ≥3. Bivariate and multivariable logistic regression analyses were performed.

**Results:**

From 1183 PTPs, 95 (8.0 %) underwent ECS. The median time to ECS was 115 min. The ECS group had higher rates of lung (27.4 % vs. 12.2 %, p < 0.001) injury, spleen (18.9 % vs. 5.5 %, p < 0.001) injury, and elevated SI (22.1 % vs. 9.8 %, p < 0.001). ECS patients had higher rates of complication (9.5 % vs. 2.1 %, p < 0.001) and death (4.2 % vs. 1.1 %, p = 0.012). Independently associated risk factors for ECS included severe head (OR 2.65, CI 1.14–6.17, p = 0.023) or abdominal (OR 2.07, CI 1.08–3.97, p = 0.028) injuries and elevated SI (OR 2.17 CI 1.25–3.79, p = 0.006).

**Conclusion:**

The national rate of ECS among PTPs presenting after MVC is 8 % with most occurring within the first 2 hours of arrival. Severe head and abdominal injuries as well as elevated SI are risk factors for ECS.

## Graphical abstract

**Figure:**
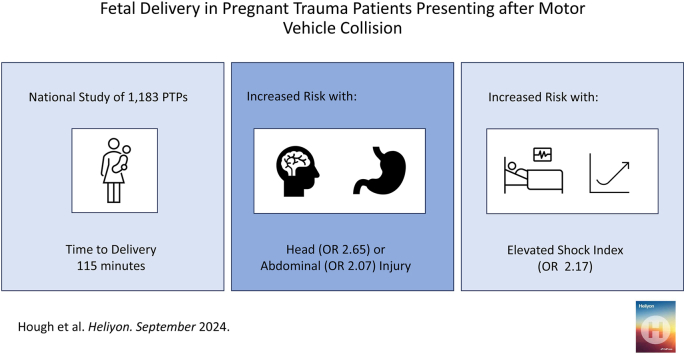

## 1 Background

Approximately 10 % of all pregnancies are affected by trauma [1] with the majority presenting after motor vehicle collision (MVC) [2,3]. Disturbingly, trauma continues to stand out as the primary driver for non-obstetric maternal morbidity and mortality [[4], [5], [6], [7], [8], [9], [10], [11], [12], [13], [14], [15], [16], [17], [18], [19]], with mortality rates in the US reaching alarming levels as high as 20 % [9]. Recent studies have attempted to characterize adverse outcomes after trauma in pregnancy, specifically after MVC. Blunt abdominal trauma (BAT) is notably linked with an escalation in preterm delivery, and a staggering 80 % of fetal deaths are associated with MVCs [[20], [21], [22], [23]].

The national rate and risk factors for emergency cesarean section (ECS) during index hospitalizations for pregnant trauma patients (PTPs) after MVC are not well established. Recognizing the predictors of ECS in PTPs is important for several reasons. Foremost, it enables trauma and obstetric teams to optimize patient management decisions and fine-tune resource allocation, ensuring vital assets like operating rooms and neonatal intensive care units are readily accessible. Moreover, it can shape public health interventions, directing them toward either preventing trauma in pregnant women or mitigating its effects. For instance, if specific injury patterns emerge as prime predictors of ECS, public safety campaigns can adapt to address these unique risks. Furthermore, understanding these predictors not only sets the stage for refining patient care strategies, but also propels further research which can improve management strategies and long-term outcomes for both mother and fetus.

The injury severity score (ISS) is an anatomic scoring system for multiple traumatic injuries and is determined using the abbreviated injury scale (AIS) score for each of the six body regions [24]. A higher ISS has been associated with increased risk of adverse fetal outcomes including fetal and neonatal loss [21,25]. While certain studies highlight no discernible difference in ISS between pregnancies with viable and nonviable outcomes [10,20], some consensus emerges around risk factors suggesting circulatory collapse such as a dwindling fetal heart rate as precursors to poor outcomes [10,[25], [26], [27], [28], [29], [30]]. One crucial metric in trauma care is the Shock Index (SI), which is the ratio of heart rate to systolic blood pressure. Elevated SI is indicative of potential hemodynamic instability, often requiring aggressive resuscitation [31]. Given its ability to depict circulatory compromise, SI could be a pivotal predictor for ECS. Compared with matched nonpregnant women in a retrospective single center level I trauma center, PTPs had lower ISS and AIS scores, however more sustained abdominal injury and suffered obstetric complications including the need for emergency delivery [32]. In this study, we investigate the national rate of ECS in PTPs presenting after MVC, hypothesizing a higher risk of ECS among those with severe injuries or elevated SI.

## 2 Methods

**Fig. 1:**
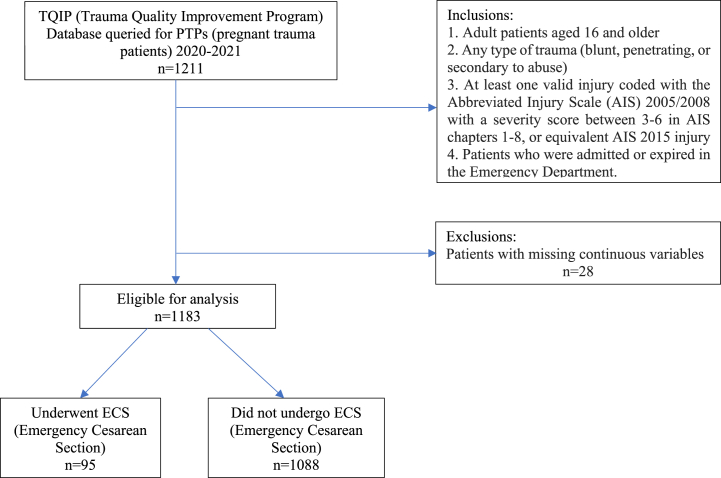
Data collection strobe (Strengthening the reporting of observational studies in epidemiology) diagram.

The Trauma Quality Improvement Program (TQIP) database was queried to identify adult PTPs from 2020 to 2021 presenting after a MVC (Fig. 1). This is a deidentified national database, and so this study was deemed exempt by our institutional review board. The TQIP database inclusion criteria are specific and include: 1. Adult patients aged 16 and older 2. Any type of trauma (blunt, penetrating, or secondary to abuse) 3. At least one valid injury coded with the Abbreviated Injury Scale (AIS) 2005/2008 with a severity score between 3 and 6 in AIS chapters 1–8, or equivalent AIS 2015 injury 4. Patients who were admitted or expired in the Emergency Department. Pregnancy was added as a comorbidity in the TQIP database starting in 2020. The primary outcome was ECS, defined by International Classification of Diseases (ICD) version-10 procedure codes: 10D00Z1, 10D00Z2, 10D20ZZ, and 10D00Z0. Two groups were compared: PTPs that underwent ECS at any point during hospitalization and PTPs that did not undergo ECS. Each delivery was performed via emergent cesarean delivery, specifically focusing on the urgent nature of these procedures in the context of trauma. There were no vaginal deliveries. Procedures such as dilation and curettage, which are typically conducted in a more controlled and semi-elective environment, were not included in our study. Our analysis centers on identifying predictors of urgent, life-saving fetal deliveries necessitated by the immediate circumstances of trauma.

Demographic variables that were collected included age and comorbidities such as hypertension, diabetes, mental health disorders, prior smoking or substance use. Presentation history such as alcohol or drug screen positivity was also collected, however, alcohol or drug screening is not universally performed for all patients and is done based on local hospital protocols. Data on blood transfusion within the first 4 h of arrival was collected as well as pre-hospital cardiopulmonary resuscitation. The injury profile consisted of the ISS and specific injury patterns including brain, lung, rib, diaphragm, liver, spleen, pancreas, stomach, small intestine, colon, rectal, bladder, and pelvis injury. Severe injury to a body region was defined by AIS grade ≥3. Vitals on arrival including heart rate (HR), respiratory rate (RR) and systolic blood pressure (SBP) were also recorded. High-risk MVC was defined by crash intrusion of the roof, driver or passenger seat greater than 12 inches or any site greater than 18 inches, ejection (partial or complete) from the automobile, death in same passenger compartment, or crash vehicle telemetry data consistent with high-risk injury. Each patient included in the analysis had a complete data set with every variable known for binary data. For continuous variables with missing data, which accounted for 2.4 % of our patient cohort, these cases were excluded from the multivariable logistic regression analysis.

Various outcomes including complications were collected including cardiac arrest, catheter associated tract infection, acute kidney injury, deep vein thrombosis, embolism, need for intubation, organ space skin or soft tissue infection, pressure ulcer, pulmonary complication, need for unplanned return to the operating room, sepsis, or ventilation associated pneumonia. Other outcomes measured were length of stay in days, overall mortality, and shock index (SI). SI ranging from 0.8 to 1.4 has been correlated with increasing mortality, transfusion needs, ISS, emergent operations, and ICU admissions in trauma patients [[33], [34], [35], [36]]. Specifically, SI ≥ 1 has also been correlated with increased transfusion needs in pregnant trauma patients and utilized in several large retrospective trauma cohort studies [[37], [38], [39], [40]]. Given these findings, and in alignment with our institutional practice supported by coauthor consensus, we defined elevated SI as ≥1.

Bivariate analyses were first performed. A Mann-Whitney-U test was used to compare continuous variables and a chi-square was used to compare categorical variables in the bivariate analysis. Categorical data was presented as percentages while continuous data was presented as a median with interquartile range. We then performed a multivariable logistic regression analysis to determine the risk of ECS. We chose these variables based on review of the literature [[6], [7], [8],^13^,^20^] and consensus among coauthors. P-values were defined as statistically significant if < 0.05. All analyses were performed with IBM SPSS Statistics for Windows (Version 29, IBM Corp., Armonk, NY). We also conducted a ROC analysis to determine the AUC for our logistic regression model.

## 3 Results

Among 1183 PTPs, 95 (8.0 %) underwent ECS. The median time to ECS was 115 min from arrival to the hospital but ranged from 16 min to 90 h. The median age was the same for the ECS and non-ECS groups (26 years old). There were no differences in comorbidities between the ECS and non-ECS groups (all p > 0.05). The ECS and non-ECS groups had a similar rate of presenting after a high risk MVC (14.7 % vs. 10.5 %, p = 0.20) and the ECS group had higher rates of blood transfusion within the first 4 h of arrival (33.7 % vs. 4.8 %, p < 0.001) (Table 1). 7 patients (0.6 %) in the non-ECS group had pre-hospital cardiopulmonary resuscitation as compared to 3 patients (3.2 %) in the ECS group.

**Table 1:** Demographics, presentation findings, and comorbidities of PTPs after MVC stratified by ECS.

Characteristic	(−) ECS	(+) ECS	p-value
Age, year, median (IQR)	26 (9)	26 (8)	0.929
Presenting history, n (%)			
High risk MVC	114 (10.5 %)	14 (14.7 %)	0.200
Alcohol screen positive	70 (9.9 %)	4 (6.3 %)	0.358
Drug screen positive	212 (42.8 %)	18 (45.0 %)	0.790
Elevated SI ≥ 1	107 (9.8 %)	21 (22.1 %)	<0.001
Transfusion∗	52 (4.8 %)	32 (33.7 %)	<0.001
Pre-hospital CPR	7 (0.6 %)	3 (3.2 %)	0.010
Diabetes	21 (1.9 %)	2 (2.1 %)	0.910
Hypertension	36 (3.3 %)	4 (4.2 %)	0.645
Mental health history	103 (9.5 %)	8 (8.4 %)	0.725
Smoking history	189 (17.4 %)	12 (12.6 %)	0.234
Substance use	114 (10.5 %)	9 (9.5 %)	0.752

PTP = pregnant trauma patient, MVC = motor vehicle collision, ECS = emergency cesarean section.

IQR = interquartile range, ADHD = attention-deficit/hyperactivity disorder.

COPD = chronic obstructive pulmonary disease.

Transfusion∗ = transfusion given within first 4 h of patient arrival.

CPR = cardiopulmonary resuscitation.

The ECS group had higher rates of overall injury burden including to the lung (27.4 % vs. 12.2 %, p < 0.001), ribs (20.0 % vs. 12.0 %, p = 0.03), diaphragm (5.3 % vs. 0.2 %, p < 0.001), spleen (18.9 % vs. 5.5 %, p < 0.001), liver (14.7 % vs. 5.2 %, p < 0.001), pancreas (3.2 % vs. 0.2 %, p < 0.001), small intestine (3.2 % vs. 0.6 %, p < 0.010), colon (5.3 % vs. 0.8 %, p < 0.001), rectum (2.1 % vs. 0 %, p < 0.001), pelvis (18.9 % vs. 9.5 %, p < 0.003), and brain (18.9 % vs. 7.9 %, p < 0.001). The ECS group also had a higher median ISS (12 vs. 5, p < 0.001) and an elevated rate of SI (22.1 % vs. 9.8 %, p < 0.001) (Table 2). ECS patients had higher rates of overall complications (9.5 % vs. 2.1 %, p < 0.001) and maternal mortality (4.2 % vs. 1.1 %, p = 0.012) (Table 3). A multivariable logistic regression analysis identified severe head injury (OR 2.65, CI 1.14–6.17, p = 0.023), elevated SI (OR 2.17 CI 1.25–3.79, p = 0.006), and severe abdominal injury (OR 2.07, CI 1.08–3.97, p = 0.028) as independently associated risk factors for ECS in PTPs presenting after a MVC (Table 4). The AUC for our logistic regression model was found to be 0.705, indicating that the model has good predictive ability (p = 0.001). These results substantiate the robustness of the identified predictors as reliable indicators for ECS in PTPs.

**Table 2:** Injury profile of PTPs after MVC stratified by ECS.

Characteristic	(−) ECS	(+) ECS	p-value
Injury Profile, n (%)			
Traumatic brain injury	86 (7.9 %)	18 (18.9 %)	<0.001
Lung	133 (12.2 %)	26 (27.4 %)	<0.001
Rib fracture	131 (12.0 %)	19 (20.0 %)	0.025
Diaphragm	2 (0.2 %)	5 (5.3 %)	<0.001
Spleen	60 (5.5 %)	18 (18.9 %)	<0.001
Liver	57 (5.2 %)	14 (14.7 %)	<0.001
Pancreas	2 (0.2 %)	3 (3.2 %)	<0.001
Stomach	0 (0 %)	1 (1.1 %)	<0.001
Small intestine	7 (0.6 %)	3 (3.2 %)	0.010
Colon	9 (0.8 %)	5 (5.3 %)	<0.001
Rectal	0 (0 %)	2 (2.1 %)	<0.001
Bladder	3 (0.3 %)	2 (2.1 %)	0.008
Pelvic fracture	103 (9.5 %)	18 (18.9 %)	0.003
ISS, Median (IQR)	5 (8)	12 (16)	<0.001

PTP = pregnant trauma patient, MVC = motor vehicle collision, ECS = emergency cesarean section.

ISS = injury severity score, IQR = interquartile range, SI = shock index.

**Table 3:** Complications and outcomes of PTPs after MVC stratified by ECS.

Characteristic	(−) ECS	(+) ECS	p-value
Complication, n (%)			
Any complication	23 (2.1 %)	9 (9.5 %)	<0.001
Cardiac arrest	3 (0.3 %)	1 (1.1 %)	0.211
CAUTI	2 (0.2 %)	1 (1.1 %)	0.107
DVT	2 (0.2 %)	1 (1.1 %)	0.107
Embolism	4 (0.4 %)	1 (1.1 %)	0.324
Intubation	6 (0.6 %)	2 (2.1 %)	0.077
Kidney	0 (0 %)	1 (1.1 %)	<0.001
Organ space SSI	1 (0.1 %)	1 (1.1 %)	0.029
Pressure ulcer	2 (0.2 %)	1 (1.1 %)	0.107
Respiratory	3 (0.3 %)	0 (0 %)	0.608
Return to OR	4 (0.4 %)	3 (3.2 %)	<0.001
Sepsis	1 (0.1 %)	0 (0 %)	0.767
VAP	3 (0.3 %)	1 (1.1 %)	0.211
LOS, days, median (IQR)	2 (3)	6 (8)	<0.001
Maternal mortality, n (%)	12 (1.1 %)	4 (4.2 %)	0.012

PTP = pregnant trauma patient, MVC = motor vehicle collision, ECS = emergency cesarean section.

CAUTI = catheter-associated urinary tract infection, DVT = deep vein thrombosis.

SSI = surgical site infection, OR = operating room, VAP = ventilator-associated pneumonia, LOS = length of stay, IQR = interquartile range.

**Table 4:** Multivariable regression analysis evaluating risk of ECS among PTPs after MVC.

Characteristic	Odds Ratio	95 % CI	p-value
Elevated SI ≥ 1	2.172	1.245–3.790	0.006
High risk MVC	1.123	0.596–2.114	0.720
Severe Injury∗			
Head	2.656	1.143–6.174	0.023
Abdomen	2.071	1.081–3.967	0.028
Thorax	1.239	0.441–3.484	0.684
Lower extremity	0.941	0.254–3.490	0.927

PTP = pregnant trauma patient, MVC = motor vehicle collision, ECS = emergency cesarean section.

SI = shock index, CI = confidence interval, ∗ = abbreviated injury scale grade ≥3.

## 4 Discussion

This study identified predictors associated with ECS in PTPs after MVC. Interestingly, while factors such as comorbidities, maternal age, and involvement in high-risk MVCs showed negligible effects on ECS rates, the prevalence of severe injuries and elevated physiological parameters were markedly connected to ECS. Specifically, severe head and abdominal injuries, coupled with an elevated SI, emerged as prime predictors of ECS.

The risk factors for ECS have not been well studied in the literature. The existing data is limited by the lower relative rates and severity of trauma in pregnancy [7,18,32,41,42]. The current study found an ECS rate of 8 %, lower compared to previous studies of pregnant patients after trauma, which have been reported to be between 11% and 30 % [7,20,23,32,43]. However, our national study specifically examined presentation after MVC. Schiff et al. performed a state-wide retrospective review of nearly 600 PTPs after MVC and concluded a ECS rate of 17 % [43], while Owattanapanich et al. studied nearly 150 PTPs after MVC at a Level I Trauma Center and found an rate of 3.5 % [32]. While the rates of ECS in pregnant patients after MVC may be relatively low, trauma has significant consequences to both the mother and fetus. ECS following trauma for patients more than 25 weeks gestation has been associated with nearly 50 % fetal death and 20 % maternal death [21]. The burden of trauma to the fetus can be significant with over a 60 % complication rate, culminating in outcomes like preterm rupture of membranes (PROM), preterm delivery, placental abruption, uterine rupture, cesarean delivery, fetal demise, and long-term disability [6,20,44].

Preterm labor and ECS in PTPs have been reported to be associated with increasing severity of trauma [[45], [46], [47], [48]]. Dalton et al. explored ISS as a predictor of maternal and perinatal outcomes, concluding ISS ≥8 to be associated with adverse maternal outcomes including ICU admission and death, and ISS ≥2 associated with short-term perinatal outcomes such as neonatal ICU admission, fetal demise, preterm birth, abruption, premature PROM, unplanned delivery and low birth weight [7]. Schuster et al. found that PTPs with ISS ≥9 had higher rates of delivery, 17 % vs. 6 % [49], and Trivedi et al. found that ISS >2 and abdominal trauma was associated with adverse perinatal outcomes [50]. The current study is consistent with previous findings with the addition of more granular data regarding specific injury patterns and the utilization of a large national database. The ECS group had higher overall injury burden compared to the non-ECS group, with higher rates of concurrent solid organ and hollow viscous injury. Previous studies suggest BAT contributes to intrauterine shear forces through acceleration-deceleration mechanisms or direct impact, potentially leading to uterine contractions, fetal distress, initiation of vaginal bleeding, membrane rupture, labor, or maternal-fetal hemorrhage and placental abruption. These mechanisms could precipitate or expedite ECS, thereby heightening perinatal risks. However, it should be considered that in our cohort, the indication for ECS could include facilitating the resuscitation and surgical treatment of severely injured mothers. Therefore, while injury severity correlates with an increased likelihood of ECS, the underlying reasons for these decisions could vary and are not exclusively linked to fetal indications [1,5,8,9,15,22,46]. It should be noted that the indications for ECS, while not specified in the TQIP database, could range from fetal distress to procedural interventions aimed at maternal care. This highlights the complexity of managing PTPs and the dual focus on both maternal and fetal health in decision-making. Thus, while severe injuries correlate with a higher likelihood of ECS, the precise reasons involve a spectrum of clinical considerations that extend beyond fetal distress alone [51].

While severe injuries to the abdomen was independently associated with increased risk of ECS, severe injuries to the head and higher median ISS were also associated. A higher injury burden can invoke a potent stress-response that may unleash a cascade of hormonal fluxes, thereby inducing hyperexcitability of the uterus and consequent contraction [5,15,21]. Given that ECS patients also have higher rates of complications and in-hospital mortality, this stress response may have been punctuated throughout their hospitalization. Regarding the association with severe head injury, there is limited data on TBI in pregnancy and its influence on the mother and fetus. However, the management of severe TBI may impact ECS in PTPs. Maternal hyperventilation or hypocarbia for intracranial pressure management may negatively affect uteroplacental perfusion [1,8] which can hasten delivery. Future research is needed to evaluate these suggested mechanisms.

SI is a metric used for prognostication and decision making in the care of trauma patients. A higher SI in adult trauma patients has been associated with predicting need for massive transfusion and correlates with higher mortality [31]. In pregnant patients, SI has been studied as a tool to predict early blood product transfusion [40] and as a parameter in indicating post-partum hemorrhage [52]. The current study found that SI was elevated in PTPs ultimately experiencing ECS compared to the non-ECS group. This has not been previously reported and serves as a quick and easy adjunct to risk-stratify ECS in PTPs involved in a MVC. There are several reasons why an elevated SI may be associated with ECS in an injured PTP. Circulatory compromise can lead to decreased perfusion of the placenta. Reduced uteroplacental perfusion can lead to fetal hypoxia, which can subsequently stress the fetus and signal the need for imminent delivery [1,5,9,53]. Furthermore, an elevated SI resulting in maternal acidosis can also contribute to fetal distress [9,[53], [54], [55]]. A hypo-perfused uterus and uterine bleeding can also result in placental insufficiency, uterine irritability and potential contractions which may instigate labor [1,5,[56], [57], [58], [59], [60]]. Lastly, an elevated SI coupled with blood loss can result in coagulopathy increasing the risk of placental abruption which poses immediate risk to the fetus and requires emergent delivery [5,8,9,15,61]. These findings are highly suggestive that severely injured patients with an elevated SI have increased risk for ECS. SI may serve as a quick tool for ECS risk stratification in PTPs after MVC. Our study also found that ECS patients, in addition to increased injury severity as indicated by ISS and SI overall, had higher rates of blood transfusion within 4 h of arrival.

The timing of ECS in PTPs presenting after any trauma has not been previously reported. This study is the first to report the median time to ECS post-trauma, revealing a sensitive window of just under 2 h. While this suggests that many ECS events may be prompted by initial assessments and urgent needs immediately following trauma, we recognize that the range of times until delivery is broad, and reasoning for time to operation is not included in the TQIP database. Therefore, while the 2-h window highlights a critical period for intervention, it should be interpreted with caution, considering the variability in clinical scenarios and the absence of specific indications for each ECS. Given the close proximity between presentation and ECS, necessity for fetal heart tracing and co-management by a multidisciplinary team for PTPs with viable pregnancies is strongly encouraged, particularly those with an elevated SI or severe injuries to the head and/or abdomen [7,27,62,63]. The literature has proposed guidelines for assessment, workup, and monitoring of PTPs, and often stresses multidisciplinary management between the emergency, trauma and obstetric services to ensure health of both the mother and fetus [3,5,11,14,16,46,[64], [65], [66], [67]].

Limitations of this study include its retrospective nature and usage of a national database which can result in errors or biases with inputting data. Though the TQIP database is quite extensive, it does not capture all trauma in the United States, and the overall population of PTPs is relatively small compared to non-PTPs. Given the strict inclusion criteria to capture significant trauma cases in the TQIP database, some PTPs in MVCs might also be underrepresented. The TQIP database also does not contain variables on gestational age which may influence delivery versus expectant management given the viability of the fetus, and does not detail cardiotocographic data or physiologic improvements in the mother after delivery. The TQIP database also does not collect variables on indications for emergency cesarean section or perinatal outcomes such as placental abruption or fetal distress which would likely affect ECS. Additionally, it is not known if any deliveries were postmortem. More prospective studies should be performed to delineate associated risk factors for ECS in PTPs, and as well as characterize their secondary outcomes such as maternal and perinatal morbidity and mortality. Regardless, this large national database study provides a contemporary rate of ECS as well as specific injury patterns seen in PTP undergoing ECS after MVC.

## 5 Conclusion and clinical implications

This national study identifies significant associations between severe injuries—particularly to the head and abdomen—and elevated SI with the likelihood of ECS in PTPs following MVCs. These associations suggest the potential for severe trauma to influence ECS decisions, though direct causality cannot be inferred. Our findings emphasize the importance of prompt and comprehensive multidisciplinary management within the first hours of hospital arrival to optimize outcomes. However, we acknowledge that these conclusions are drawn from correlational data, and the lack of specific indications for ECS is a limitation that could affect the interpretation of these risk factors. Moreover, these findings magnify the potential of SI as a swift, straightforward tool for risk stratification in this patient population, advocating for its wider consideration in the initial assessment of PTPs post-MVC. Further research may focus on refining predictive models and exploring strategies to mitigate risks, enhancing clinical practices, and possibly sculpting preventive measures, with an aim to curtail the traumatic impact on PTPs.

## Funding/Support

The authors received no funding for this work.

## Conflicts of Interest/Disclosure

The authors have no relevant financial disclosures.

## Ethics and consent

The TQIP (Trauma Quality Improvement Program) database is deidentified national database and deemed exempt for ethics approval by the University of California, Irvine Institutional Review Board. Waiver of consent was granted given our study is retrospective and uses a deidentified database.

## CRediT authorship contribution statement

**Michelle Hough:** Conceptualization, Data curation, Formal analysis, Investigation, Methodology, Project administration, Writing – original draft, Writing – review & editing. **Jeffry Nahmias:** Conceptualization, Data curation, Formal analysis, Investigation, Methodology, Supervision, Validation, Visualization, Writing – review & editing. **Jeffrey Santos:** Conceptualization, Data curation, Supervision, Validation, Visualization, Writing – original draft, Writing – review & editing. **Lourdes Swentek:** Conceptualization, Supervision, Validation, Visualization, Writing – review & editing. **Robert Bristow:** Conceptualization, Supervision, Validation, Visualization, Writing – review & editing. **Jennifer Butler:** Conceptualization, Supervision, Validation, Visualization, Writing – review & editing. **Areg Grigorian:** Conceptualization, Data curation, Formal analysis, Investigation, Methodology, Project administration, Resources, Software, Supervision, Validation, Visualization, Writing – original draft, Writing – review & editing.

## Declaration of competing interest

The authors declare that they have no known competing financial interests or personal relationships that could have appeared to influence the work reported in this paper.
